# Investigation of high-pressure planetary ices by cryo-recovery. I. An apparatus for X-ray powder diffraction from 40 to 315 K, allowing ‘cold loading’ of samples

**DOI:** 10.1107/S1600576718003965

**Published:** 2018-04-27

**Authors:** Ian G. Wood, A. Dominic Fortes, David P. Dobson, Weiwei Wang, Lucjan Pajdzik, John Cosier

**Affiliations:** a Department of Earth Sciences, University College London, Gower Street, London WC1E 6BT, UK; b ISIS Facility, STFC Rutherford Appleton Laboratory, Harwell Science and Innovation Campus, Chilton, Oxfordshire OX11 0QX, UK; c Oxford Cryosystems Ltd, 3 Blenheim Office Park, Long Hanborough, Oxford OX29 8LN, UK

**Keywords:** X-ray powder diffraction, low temperatures, cold loading, planetary ices

## Abstract

A low-temperature stage for X-ray powder diffraction in Bragg–Brentano reflection geometry is described. The temperature range covered is 40–315 K, with a temperature stability at the sample within ±0.1 K of the set point. The stage operates by means of a Gifford–McMahon (GM) closed-cycle He refrigerator; it requires no refrigerants and so can run for an extended period (in practice at least 5 d) without intervention by the user. The sample is cooled both by thermal conduction through the metal sample holder and by the presence of He exchange gas, at ambient pressure, within the sample chamber; the consumption of He gas is extremely low, being only 0.1 l min^−1^ during normal operation. A unique feature of this cold stage is that samples may be introduced into (and removed from) the stage at any temperature in the range 80–300 K, and thus materials which are not stable at room temperature, such as high-pressure phases that are recoverable to ambient pressure after quenching to liquid nitro­gen temperatures, can be readily examined. A further advantage of this arrangement is that, by enabling the use of pre-cooled samples, it greatly reduces the turnaround time when making measurements on a series of specimens at low temperature.

## Abstract 2

A low-temperature stage for X-ray powder diffraction in the range 40–315 K is described. A unique feature of the apparatus is that samples may be introduced into the stage (and removed from it) at any temperature above 80 K.

## 1. Introduction   

This paper describes a cryostat for X-ray powder diffraction, designed in such a way that it allows samples to be loaded (and removed) at any temperature between 80 and 300 K. We believe that this apparatus should be of great value in many areas of research, for example when studying compounds that are liquids or gases at room temperature but which may be crystallized by freezing in liquid nitro­gen, or when examining materials in which solid-state phase transformations may be inhibited at low temperature. In particular, our own research programme on the structures and physical properties of planetary ices frequently involves studying materials that melt or decompose far below 300 K. These materials may be divided into two classes, those which are stable at atmospheric pressure (*P*) and low temperature (*T*) and those which are truly stable only at high pressure, but which may be recovered metastably to ambient pressure if quenched to ∼80 K. Examples of the first class of materials include the mineral meridianiite (MgSO_4_·11H_2_O; Peterson & Wang, 2006 ▸; Fortes *et al.*, 2008 ▸), which melts incongruently at 275 K to a mixture of epsomite (MgSO_4_·7H_2_O) and brine, and related compounds obtained by the substitution of Mg by transition metal cations (Fortes *et al.*, 2012*a* ▸,*b* ▸, 2017 ▸). Similarly, compounds such as the low-pressure cubic phase of ammonia dihydrate (ADH I; see *e.g.* Fortes *et al.*, 2003 ▸), which melts at 176 K and which must be crystallized by temperature cycling across the melting point (Bertie & Shehata, 1984 ▸), fall into this class. Even more extreme examples are provided by compounds such as carbon dioxide, methane and butane, which are gaseous at atmospheric pressure and room temperature but crystalline at liquid-nitro­gen temperatures. The second class of these materials includes the high-pressure phases of water ice itself; for example, ice III, which is stable only in a very small region of *P*/*T* space (∼0.2 < *P* < ∼0.35 GPa; ∼240 < *T* < ∼255 K), will not transform into ice II on cooling provided the cooling rate is greater than about 1 K min^−1^ (although some proton ordering may occur; see *e.g.* Knight & Singer, 2006 ▸) and so may be recovered to ambient pressure at 80 K. Similarly, as described in our accompanying paper (Wang *et al.*, 2018 ▸), we have found that a high-pressure phase of MgSO_4_·5H_2_O, formed by decomposition of epsomite at ∼1.8 GPa, may be recovered by quenching at ∼80 K and then examined by X-ray diffraction outside the pressure cell.

For both of the classes of materials described above, neutron powder diffraction probably provides both the best and experimentally the most straightforward method by which to determine their crystal structures and thermal expansion coefficients. In addition to the greater suitability of neutron scattering for the determination of the positions of light elements, the relatively large size of the sample environments for low-temperature neutron powder diffraction means that samples may readily be ground under liquid nitro­gen, transferred to a sample holder that has been pre-cooled to ∼80 K and then introduced into a cryostat without significant warming (see *e.g.* Fortes *et al.*, 2010 ▸). There are, however, several difficulties associated with such experiments. Firstly, the availability of suitable high-resolution neutron powder diffractometers is limited. Secondly, especially for diffractometers at spallation sources operating by the time-of-flight method, the accessible *d*-spacing range may not extend to sufficiently large spacings to allow indexing of the diffraction patterns, especially as many of these compounds are of low symmetry. Thirdly, although neutron powder diffraction with protonated samples is possible (*e.g.* Wilson *et al.*, 2014 ▸), because of the large incoherent cross section of hydrogen it is normally necessary to work with perdeuterated ‘analogue’ samples rather than their naturally occurring protonated isotopologues.

Clearly, in these circumstances, angle-dispersive X-ray powder diffraction has advantages of ready access, high resolution extending to large *d* spacings, and the ability to measure both protonated and deuterated samples without detriment (albeit with little or no ability to determine H/D positions). However, although a number of cold stages for reflection geometry Bragg–Brentano parafocusing X-ray powder diffractometers are currently available commercially, some of which are capable of reaching temperatures as low as 12 K, these stages suffer from the disadvantage that they are generally designed to be loaded with the sample at room temperature and thus it is impossible, or at best extremely difficult, to use them to study materials which melt or decompose substantially below 300 K. A further disadvantage of such apparatus is that the sample is usually held within the evacuated part of the stage. This simplifies construction and gives the highest X-ray transmission, as only one X-ray transparent window is required, but it also results in the sample temperature being less well determined; as there is no exchange gas present, the sample surface, which is cooled only by conduction of heat through the sample, may not be in equilibrium with the temperature sensor. Furthermore, since the vacuum space on such stages is usually continuously pumped, there is the possibility that large amounts of the sample may be lost by sublimation, especially at temperatures approaching the melting point. To try to address these deficiencies, we have described previously a low-temperature stage for X-ray powder diffraction which operates using thermoelectric cooling (Wood *et al.*, 2012 ▸). This satisfied the requirements that the sample could be loaded ‘cold’ and that the sample was not under vacuum, but only at the cost of restricting the temperature range to that above ∼245 K. In this paper we describe a ‘cold-loadable’ low-temperature stage for X-ray powder diffraction in Bragg–Brentano reflection geometry with very much better cryogenic performance. So as to make the stage suitable for accurate measurements of lattice parameters, further requirements were that the sample temperature should be stable and accurately known, with minimal temperature gradients across the sample, and that the sample position should vary as little as possible on changing temperature. The temperature range covered by the new apparatus is 40–315 K, with temperature stability at the sample within ±0.1 K of the set point; the change in sample position is less than 0.1 mm across the full temperature range (see below). Samples may be introduced into (and removed from) the stage at any temperature in the range 80–300 K. The stage operates by means of a Gifford–McMahon (GM) closed-cycle He refrigerator, requiring no refrigerants, and so can run effectively indefinitely without intervention by the user. The sample is cooled both by thermal conduction through the sample holder and by the presence of He exchange gas (at ambient pressure) within the sample chamber; the consumption of He gas is extremely low, being only 0.1 l min^−1^ during normal operation.

## 2. Construction and cryogenic performance of the apparatus   

**Figure 1:**
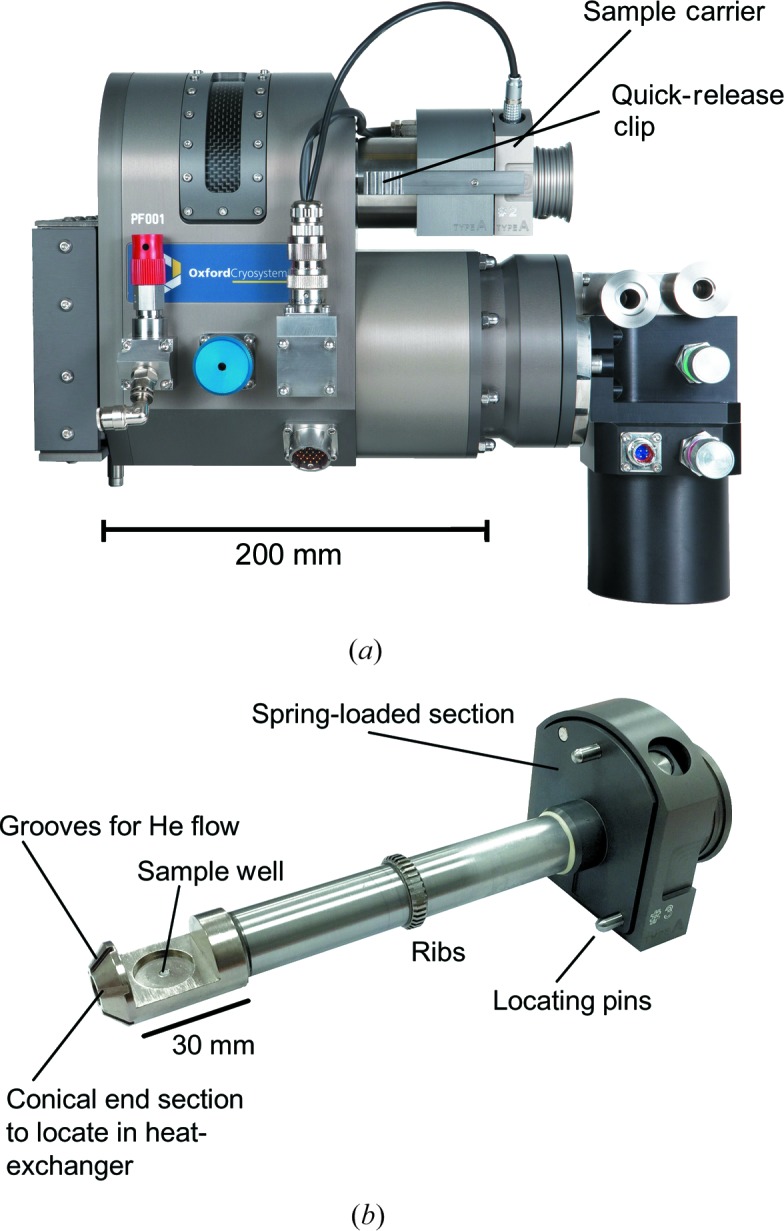
(*a*) The complete PheniX-FL low-temperature stage. (*b*) The sample carrier.

The stage, shown in Fig. 1 ▸ and termed a PheniX Front Loader (PheniX-FL) by the manufacturers (Oxford Cryosystems Ltd, Oxford, UK), is a highly modified version of the Oxford Cryosystems PheniX cryostat. It is designed to be mounted on a Bragg–Brentano geometry parafocusing powder diffractometer (such as a PANalytical X’Pert Pro) in which the counter arm rotates in the vertical plane. As in the standard PheniX, a GM closed-cycle refrigerator (CCR, but single stage in this case, rather than two stage) is mounted within the body of the apparatus; this CCR operates using compressed helium gas provided by a water-cooled Oxford Cryosystems Cryodrive compressor. The cold head of the CCR is thermally linked to a cylindrical heat-exchanger block, centred on the axis of the stage. This heat exchanger, formed from nickel-plated copper, has a conical depression machined in it so as to mate with the conical end of the sample carrier (sometimes referred to as a ‘centre stick’ – see below). A cylindrical sample access tube, with an aluminized Mylar window subtending an angle of 200°, to allow passage of the incident and diffracted X-ray beams, is connected to the heat exchanger and to the body of the stage, so as to allow the vacuum space of the stage to be continuously evacuated by means of a turbomolecular vacuum pump; the outer window of the stage is formed from carbon fibre. The whole of the sample carrier, which fits within the access tube, thus remains outside the vacuum space of the cryostat. The ‘cold end’ of the sample carrier (Fig. 1 ▸ *b*) is constructed from nickel-plated copper, terminated in a grooved truncated cone so as to mate with the heat exchanger and allow He flow, after which there is a semi-cylindrical section which contains the sample well. The central tubular part of the sample carrier, which connects the warm and cold ends, is made from stainless steel tube, foam-filled to reduce convection; a set of raised copper ribs half-way along this stainless steel tube rubs on the inside of the sample access tube at the point at which it is joined to the radiation shield of the cryostat so as to provide thermal contact. The sample to be examined is pressed into a cylindrical well in the sample carrier, 16 mm in diameter and 1.5 mm deep, and is then introduced into the cold stage *via* the sample access tube. We have found this sample size to be convenient for our PANalytical X’Pert Pro powder diffractometer, as it allows a 10 × 10 mm illuminated area of the sample to be employed (these sample dimensions correspond closely to those of one of the standard holders supplied with the instrument).

The sample carrier is held in place by quick-release spring clips on the body of the stage at the warm end of the access tube, which remains at room temperature throughout. A spring contained within the sample carrier (also at the warm end) ensures that the conical cold end is pressed tightly against the conical mating surface of the heat-exchanger block, ensuring good thermal contact between the sample carrier and heat exchanger and also ensuring that the sample is located accurately on the axis of the stage (and thus on the axis of the powder diffractometer). Rotation of the sample carrier is prevented by three round-ended pins, also at the warm end of the stage, which locate snugly into holes in the body of the stage. As in all experiments using Bragg–Brentano reflection geometry, if accurate cell parameters are to be obtained, it is essential that the sample position is determined in the data analysis, which may require the sample to be mixed with a standard material before loading. Here, the stability of the specimen position in the PheniX-FL as the sample temperature changes was determined from Rietveld refinements of diffraction patterns from four samples of cubic materials, Fe_1−*x*_Ni_*x*_Si mixed with MgO (with *x* = 0, 0.1, 0.2; Hunt *et al.*, 2017 ▸) and LaB_6_ mixed with NBS (NIST) SRM640 Si (unpublished), using the *GSAS* suite of programs (Larson & Von Dreele, 2000 ▸) with the *EXPGUI* graphical interface (Toby, 2001 ▸). All four experiments showed consistent behaviour. The refined values of the specimen displacement parameter indicated that the position of the sample, along a line perpendicular to its surface, moved upwards by about 60 µm over the temperature range from 40 to 120 K, after which there was a smaller, more gradual, downward dis­place­ment of about 20 µm, from 120 to 300 K.

So as to allow sample carriers to be exchanged whilst the stage is cold, there is no seal between the sample carrier, the access tube and the atmosphere. Instead, at temperatures below 285 K, a small amount of He gas is flowed continuously through the stage. This gas enters the sample space on the axis of the heat-exchanger block; it then flows through six radial grooves in the conical end of the sample carrier and finally passes along the sample access tube, between the wall of the tube and the shaft of the sample carrier, before it exits the cold stage by the warm end of the sample carrier. In operation, the sample thus sits in a flow of cold He gas at atmospheric pressure. Normally, the He flow rate is only 0.1 l min^−1^, allowing the stage to operate continuously for roughly 70 d from a single cylinder of helium. Upon exchanging samples when cold, however, the He flow rate is momentarily increased to 1.0 l min^−1^ to prevent ingress of the laboratory atmosphere. The temperatures of both the heat-exchanger block and the cold end of the sample carrier are measured using DT-670 silicon diode thermometers supplied by Lake Shore Cryotronics Inc., which prior to installation in the PheniX-FL were calibrated by Oxford Cryosystems against another DT-670 diode and a Lake Shore RhFe resistance thermometer (both of which had previously been calibrated by Lake Shore).

For compatibility with various X-ray powder diffractometers and their surrounding radiation enclosures, construction of the stage is necessarily compact, with the distance between the room-temperature end of the removable sample carrier and the centre of the cold sample being only 140 mm. This inevitably results in a higher base temperature (40 K) than is obtained in a standard Oxford Cryosystems PheniX stage (12 K). The cool-down time of the PheniX-FL from room temperature to 80 K is about 75 min; to maintain good temperature control on cooling below 80 K we have found it best then to reduce the temperature by no more than 1 K min^−1^.

**Figure 2:**
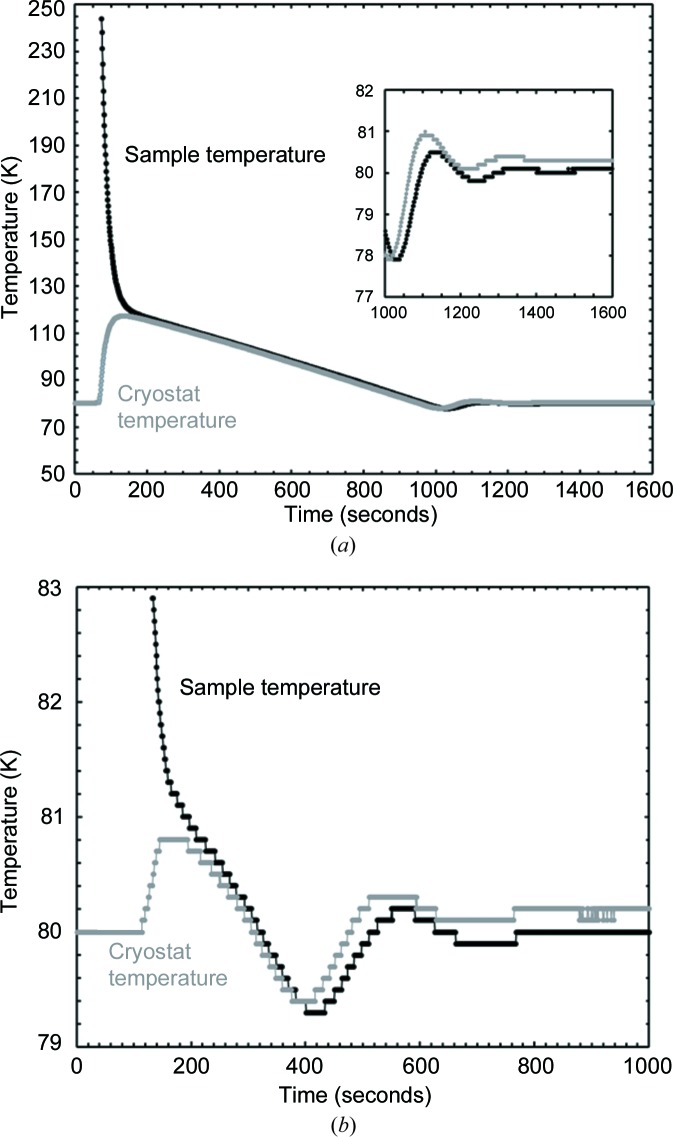
Temperature logs (at 1 s intervals) when, with the cryostat running at 80 K, (*a*) a sample at room temperature is inserted and (*b*) a sample pre-cooled to ∼80 K is inserted.

The stage is controlled through a bespoke digital controller, which allows the sample temperature to be ramped at a set rate, held constant for a set time *etc*. This controller also sets the helium gas flow rate, which can be increased, by pressing a ‘Purge’ button, from the normal 0.1 l min^−1^ to 1 l min^−1^ for a period of 60 s to allow the sample to be exchanged. The controller reads the temperature of the two diode thermometers, one mounted on the sample carrier in a recess underneath the centre of the sample and the other mounted in the heat-exchanger block of the cryostat, and automatically switches to the latter when samples are exchanged and the sample carrier diode is disconnected. The temperature control algorithm is of the three-term (proportional–integral–differential) type and the temperature control is to within ±0.1 K of the set point throughout the whole of the operating range. In normal operation the temperature control is with respect to that of the sensor in the sample carrier, but this control switches automatically to the heat-exchanger block when the sample carrier is removed. When the system is controlling at a stable temperature, the temperature offset between the heat-exchanger block and the sample carrier is typically 0.2 K (Fig. 2 ▸). At the end of the experiment, an ‘End’ button switches off the cooling, warms the stage to 310 K and continues to flow helium gas through the stage at 310 K for 30 min to remove any residual moisture, after which the system switches off completely. Data logging of the sample temperature, cryostat temperature and set point is easily accomplished after connection of the controller to a computer using the *CryoConnector* software supplied by the manufacturer.

The accuracy of the measurement of the sample temperature in the PheniX-FL was investigated by determining the temperatures at which samples of ethanol and water melted (expected melting temperatures 159.01 and 273.15 K, respectively; Rumble, 2018 ▸). For ethanol, to avoid absorption of water when preparing the sample, liquid from a freshly opened bottle of absolute ethanol (≥99.8%, AnalaR NORMAPUR ACS, supplied by VWR Chemicals; water content stated to be <0.1%) was pipetted into the well of the sample carrier at room temperature, and the carrier was then put immediately into the PheniX-FL with the stage running at 120 K. On warming to 140 K the diffraction pattern of crystalline ethanol was observed. The sample was then slowly warmed, allowing equilibration for 10 min at each temperature before measuring the diffraction pattern. It was found that the onset of melting (as judged by a small decrease in the heights of the Bragg reflections and a small increase in background) began at 158.0 K; at 158.5 K, the sample was clearly composed of both solid and liquid; at 159.0 K, no Bragg reflections were seen and the sample was entirely liquid. For water (18.2 MΩ resistivity water from an Elga Scientific DV35 purification system), the sample was prepared in a cold room at ∼260 K by loading powdered ice into the sample carrier, which was then put into the PheniX-FL with the stage running at 250 K. In this case, no evidence of melting was detected at 272.5 K; at 273.0 K both solid and liquid were present, but at 273.5 K the sample was entirely liquid. Bearing in mind that any contamination of the ethanol by water will be expected to reduce the melting temperature by ∼1 K per wt% H_2_O, we can conclude from these experiments that the error in the absolute sample temperature was, at most, about 1 K and similarly that the temperature gradient across the sample was also, at most, about 1 K.

Exchange of samples at low temperature is very easy and rapid. All that is required is to unplug the temperature sensor cable from the sample carrier that is in the stage at the time; increase the helium flow (Purge button); slowly remove the sample carrier from the access tube (taking ∼5 s in total to prevent excessive ingress of air from the laboratory); insert the new sample carrier; and reconnect the temperature sensor cable. This process can be carried out at any temperature, though it is considered inadvisable to do so below 80 K as this may result in condensation of atmospheric air in the apparatus. The robust nature of the control system means that it is not necessary for the sample carrier that is inserted into the stage to be pre-cooled to the temperature of the heat-exchanger block. Fig. 2 ▸(*a*) shows the sample and cryostat heat-exchanger temperature profiles when a sample carrier, initially at room temperature, is put into the stage running at 80 K; it can be seen that the sample temperature has reached 120 K within 2 min and 100 K after about 8 min; after 28 min the sample temperature is within ±1 K of the 80 K set point and by about 31 min it is within ±0.1 K. Fig. 2 ▸(*b*) shows the more normal situation when a sample carrier that has been pre-cooled in liquid nitro­gen is loaded. In this case, control to within ±0.1 K of the 80 K set point is achieved within 8 min. Even with samples that are stable at room temperature, this system does, therefore, offer a considerable advantage in turnaround time when compared with a conventional cold stage. For example, if it were desired to collect data from a set of samples at 80 K using a standard Oxford Cryosystems PheniX, the turnaround time would be of the order of 90 min, as the stage would have to be warmed to room temperature and then cooled again every time the sample was changed; in the present case, if samples are pre-cooled in liquid nitro­gen, the turnaround time is reduced to under 10 min.

## 3. Example X-ray data   

Examples of the use of this apparatus to study cold-loaded samples of high-pressure planetary ices quenched into liquid nitro­gen are given in the accompanying paper, in which our high-pressure apparatus is also described (Wang *et al.*, 2018 ▸). We confine the discussion here mainly to considerations of factors such as the angular range available, the transmission of the cryostat windows and a method by which inadvertent incorporation of ice into the sample, from condensation of atmospheric water vapour, can be minimized.

The construction of the cold stage places no additional restrictions on the angular range of the diffraction pattern that may be examined above those resulting from the mechanical limits of our PANalytical X’Pert Pro powder diffractometer. The outer window of the stage, which extends below the plane of the sample, is supported by a metal rib that subtends an angle of ±5.5° from a line perpendicular to the sample surface on a radius of 60 mm. In standard Bragg–Brentano para­focusing reflection geometry, with a 10 mm irradiated sample length and a diffractometer radius of 240 mm, measurements may, therefore, be made over the angular range 0 < 2θ < 162° without the window support impinging on either the incident or diffracted beams. The width of the window is 18 mm, slightly greater than the maximum dimension of the sample and substantially greater than the illuminated sample width (∼10 mm) if a square of illumination is used. The inner window has no supporting rib and imposes no additional restrictions on the accessible angular range. The transmission of the windows was determined from measurements of samples of 0.3 µm Lindé α-Al_2_O_3_ in air and in the cold stage at room temperature and was found to be 34% for Co *K*α_1_ radiation; with the assumption that the windows are composed entirely of carbon, this would rise to ∼51% for Cu *K*α and ∼92% for Mo *K*α. It would, of course, be possible to improve the transmission by the use of thinner windows, but only at the cost of making them less robust; in practice, we have found the current arrangement to be adequate, even with Co *K*α_1_ radiation.

**Figure 3:**
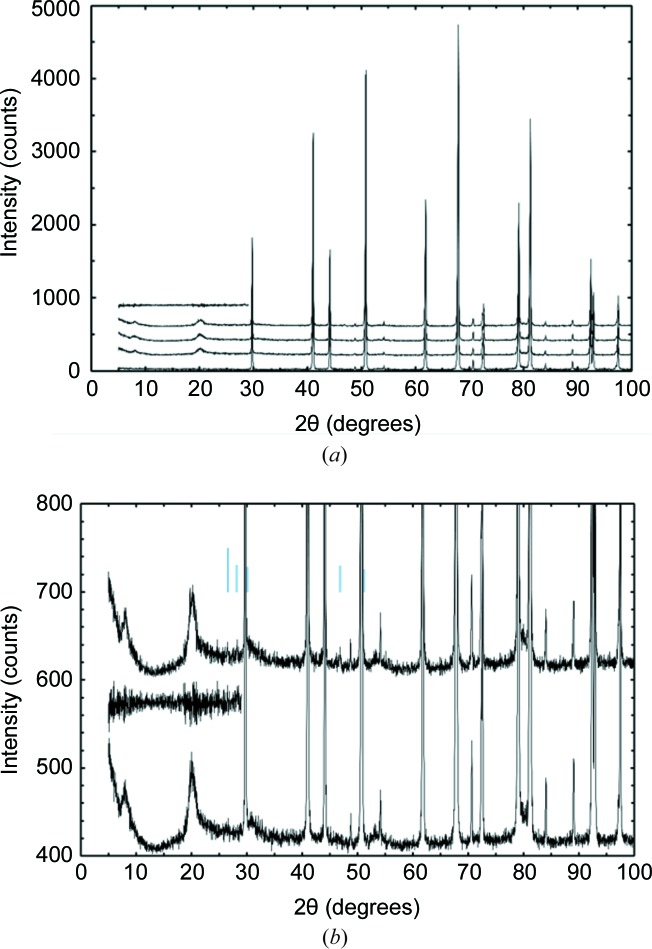
(*a*) Diffraction patterns from 0.3 µm Lindé α-Al_2_O_3_ (Co *K*α_1_, 40 kV, 30 mA, incident-beam Ge(111) monochromator, 10 × 10 mm illuminated sample area). Reading from the bottom up: (i) in a standard sample holder, *i.e.* outside of the cold stage; (ii) in the cold stage at room temperature; (iii) at 80 K, with the sample loaded at room temperature [temperature profile on loading shown in Fig. 2 ▸(*a*)]; (iv) at 80 K, with the sample immersed in liquid nitro­gen and loaded into a sample carrier that had also been cooled in liquid nitro­gen [temperature profile on loading shown in Fig. 2 ▸(*b*)]; and (v) the difference between (iv) and (iii). The same counting time (191 min) was used for (ii)–(iv); pattern (i), which was counted for 53 min, has been scaled to give approximately the same peak heights in the figure. Patterns (ii)–(v) have been offset vertically for clarity. (*b*) As for panel (*a*), but with the vertical scale increased and only patterns (iii), (iv) and their difference (v) shown. The position of the five strongest reflections from ice I*h* (two of which are partially overlapped by reflections from alumina) are marked as vertical bars, with heights proportional to the intensities given in ICDD (International Centre for Diffraction Data; http://www2.fiz-karlsruhe.de/icsd_home.html) database entry 01-085-0857, after Dowell & Rinfret (1960 ▸).

A potential difficulty inherent in cold-loading samples is that they may become contaminated with ice crystals introduced by condensation of water vapour from the laboratory atmosphere. In many of our planetary ice samples, such as those discussed in the accompanying paper (Wang *et al.*, 2018 ▸), water is an intrinsic component of the sample and it is therefore difficult to be certain, either by visual inspection (since the materials are, for the most part, white in colour) or by examination of the diffraction patterns, whether the sample preparation has introduced additional surface icing. We therefore used 0.3 µm Lindé α-Al_2_O_3_ as a test sample here to investigate both the performance of the cold stage and the degree to which contamination of the sample surface by condensed ice can be minimized. Fig. 3 ▸ shows four diffraction patterns from this material: (i) in a standard sample holder, *i.e.* outside of the cold stage; (ii) in the cold stage at room temperature; (iii) with the sample loaded at room temperature and then rapidly cooled to 80 K [the temperature profile for this loading is plotted in Fig. 2 ▸(*a*)]; and (iv) with the sample prepared in liquid nitro­gen and loaded into a sample carrier that had also been cooled in liquid nitro­gen [temperature profile on loading shown in Fig. 2 ▸(*b*)]. For variant (iv), the following procedure was adopted to prepare the sample. Firstly, the PheniX-FL sample carrier (with the tubular shaft wrapped in aluminium foil) and a cryo-mortar were precooled at ∼253 K in a cold room (although not essential, the use of a cold room greatly reduces the absolute ambient humidity). The cryo-mortar and PheniX-FL sample carrier were then cooled with liquid nitro­gen until they reached ∼80 K and could contain liquid N_2_ with only very gentle boil off. As it did not require further grinding, the Al_2_O_3_ sample was then put into the cryo-mortar and stirred in liquid N_2_, after which it was transferred to the PheniX-FL sample carrier using a stainless steel spoon and spatula that had also been cooled to ∼80 K. Finally, to prevent contact with the atmosphere, a steel plate (cooled to ∼80 K) was placed over the sample, the cold end of the sample carrier was put into a pre-cooled glass tube and the sample carrier was taken to the X-ray laboratory with this tube partially immersed in liquid N_2_. To minimize contact with the atmosphere as the sample was loaded into the cold stage on the diffractometer, the aluminium foil and the steel cover plate were then quickly removed from the sample carrier just before it was put into the access tube.

Examination of Fig. 3 ▸(*a*) shows that the main additional features in the diffraction pattern arising from the sample environment are the two broad peaks from the cryostat windows at 2θ ≃ 8° and 2θ ≃ 20°. Although these peaks are not negligible in intensity, we consider them acceptable; for example, the height above background of the peak at 2θ ≃ 20°, which is the stronger of the two, is less than 2% of the height of the strongest reflection from α-Al_2_O_3_ (about 70 counts, compared with 3600). Furthermore, this background may be removed, or very nearly removed, by subtraction of a diffraction pattern collected from a standard material, ideally of comparable scattering density to the sample, which does not give Bragg reflections below 2θ ≃ 25°. This is illustrated by the uppermost trace in Fig. 3 ▸(*a*), which gives the intensity difference between the two diffraction patterns collected at 80 K.

The degree to which contamination of the sample by ice can be avoided when cold loading is best seen in Fig. 3 ▸(*b*), which shows the data sets collected at 80 K from warm-loaded and cold-loaded samples, with a magnified vertical scale; the positions of the five strongest reflections from ice I*h* are marked by vertical blue bars. The 110 reflection from ice I*h* (2θ = 46.9°) is the most visible ice peak, but its height is only ∼20 counts above the background, and so only ∼0.5% of the height of the strongest peak from α-Al_2_O_3_. On careful examination of the pattern of the cold-loaded sample, the 100 (2θ = 26.6°) and 002 (2θ = 28.2°) reflections are also found to be just visible. It should, perhaps, be mentioned that the sample preparation and loading at 80 K shown in Fig. 3 ▸ were not done especially quickly, and so the amount of ice present can be regarded as typical rather than representing the smallest amount that can be achieved.

**Figure 4:**
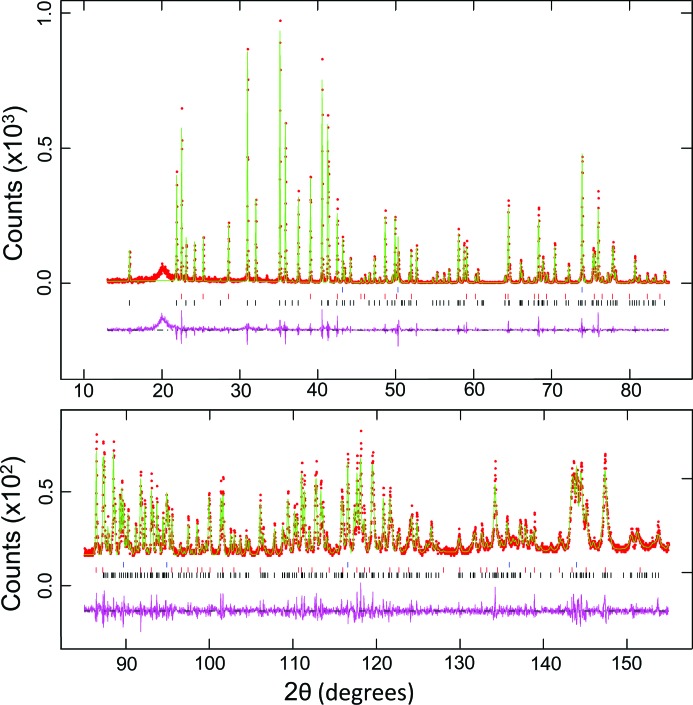
Le Bail refinement of a sample consisting of a mixture of MgGeO_3_ (both clinopyroxene and ilmenite polymorphs) and MgO at 60 K. The data were collected with Co *K*α_1_ radiation (40 kV, 30 mA) and an incident-beam Ge(111) monochromator. The total scan time was 120 min. A constant area of the sample of 10 × 8.5 mm was illuminated and the data were then corrected to fixed-divergence slit geometry using software supplied by the manufacturer of the diffractometer (PANalytical). Observed data points are shown in red, the calculated diffraction pattern in green and the difference curve in pink. The reflection markers are for (from the bottom up) MgGeO_3_, clinopyroxene phase; MgGeO_3_, ilmenite phase; MgO.

In the accompanying paper (Wang *et al.*, 2018 ▸) we give two further examples of the use of the PheniX-FL with cold loading to study high-pressure planetary ice phases that have been recovered to atmospheric pressure at 80 K. Samples of this type are more difficult to prepare than materials that are stable at ambient pressure and, as they exist only metastably (in some cases being well outside their stability field), their diffraction patterns may show line broadening. In the present paper, therefore, as a final example of the use of this cold stage with a well crystallized material, we have chosen a data set collected at 60 K from a sample composed of a mixture of MgGeO_3_ (in both the clinopyroxene and ilmenite polymorphs) and MgO; in this case the sample was loaded at room temperature. Fig. 4 ▸ shows a Le Bail refinement (Le Bail *et al.*, 1988 ▸) of these data made using the *GSAS* suite of programs with the *EXPGUI* graphical interface. The following lattice parameters were obtained: MgGeO_3_ (clinopyroxene), space group *C*2/*c*, *a* = 9.5903 (1), *b* = 8.9233 (1), *c* = 5.15472 (7) Å, β = 100.898 (1)°, *V* = 433.17 (1) Å^3^; MgGeO_3_ (ilmenite), space group 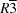, *a* = 4.93236 (7), *c* = 13.7237 (2) Å, *V* = 289.143 (8) Å^3^; MgO, space group 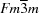, *a* = 4.20733 (6) Å, *V* = 74.477 (3) Å^3^. The χ^2^ value for the fit was 1.09, with weighted and unweighted profile *R* factors (including background) of 0.2499 and 0.1604, respectively (if the region 17.7 < 2θ < 21.4°, containing the strong ‘window peak’, is excluded from the fit, the χ^2^ and profile *R* factors drop to 0.69, 0.20 and 0.13, respectively). Further examples of the use of the apparatus for high-precision studies of the thermal expansion of materials that are stable at room temperature and pressure (Fe_1−*x*_Ni_*x*_Si and gold) can be found in the papers by Hunt *et al.* (2017 ▸) and Pamato *et al.* (2018 ▸), respectively.

The PheniX-FL has now been in operation in our laboratory for over three years and, although it is still essentially a prototype, we have encountered very few problems when using it. When compared with a standard PheniX stage (or other similar CCR-based sample environments) the main disadvantages of the PheniX-FL are its higher base temperature and the increased absorption of the X-ray beam by the additional window. At the very lowest temperatures (40–50 K) we have sometimes encountered contamination of the sample (probably confined to its surface) by solid nitro­gen and CO_2_, but the Bragg reflections from these contaminants (which can be removed by warming) are relatively weak and few in number and so have not caused any problems in our data analysis. Occasionally, we have found that the flow of helium purge gas becomes partially blocked (possibly from formation of ice at the point where the ribs of the sample carrier make mechanical contact with the access tube to heat-sink the sample carrier to the radiation shield of the cryostat) but this has never reached the point at which it would cause failure of the experiment, even during periods of continuous operation of up to 5 d. In comparison with these disadvantages, unless the ability to access temperatures below 40 K is essential, we feel that the PheniX-FL stage has many significant benefits, such as its cold-loading capability, the rapidity with which samples may be exchanged and the fact that the sample sits in an atmosphere of helium exchange gas. This latter feature suggests that the system could be modified without too much difficulty to accept alternative sample mounts, such as samples in glass capillary tubes.
